# EPGNet: Enhanced Point Cloud Generation for 3D Object Detection

**DOI:** 10.3390/s20236927

**Published:** 2020-12-04

**Authors:** Qingsheng Chen, Cien Fan, Weizheng Jin, Lian Zou, Fangyu Li, Xiaopeng Li, Hao Jiang, Minyuan Wu, Yifeng Liu

**Affiliations:** 1 School of Electronic Information, Wuhan University, Wuhan 430072, China; 2019202120064@whu.edu.cn (Q.C.); jwz@whu.edu.cn (W.J.); zoulian@whu.edu.cn (L.Z.); 2019202120065@whu.edu.cn (F.L.); xiaopengli2014@whu.edu.cn (X.L.); jh@whu.edu.cn (H.J.); wmy@whu.edu.cn (M.W.); 2 National Engineering Laboratory for Public Safety Risk Perception and Control by Big Data (NEL-PSRPC), Beijing 100041, China; liuyifeng3@cetc.com.cn

**Keywords:** 3D objection detection, symmetry, enhanced point cloud, autonomous driving

## Abstract

Three-dimensional object detection from point cloud data is becoming more and more significant, especially for autonomous driving applications. However, it is difficult for lidar to obtain the complete structure of an object in a real scene due to its scanning characteristics. Although the existing methods have made great progress, most of them ignore the prior information of object structure, such as symmetry. So, in this paper, we use the symmetry of the object to complete the missing part in the point cloud and then detect it. Specifically, we propose a two-stage detection framework. In the first stage, we adopt an encoder–decoder structure to generate the symmetry points of the foreground points and make the symmetry points and the non-empty voxel centers form an enhanced point cloud. In the second stage, the enhanced point cloud is input into the baseline, which is an anchor-based region proposal network, to generate the detection results. Extensive experiments on the challenging KITTI benchmark show the effectiveness of our method, which has better performance on both 3D and BEV (bird’s eye view) object detection compared with some previous state-of-the-art methods.

## 1. Introduction

In recent years, 3D object detection has been widely investigated by the industry and academia thanks to its applications in various fields, such as autonomous driving and robotics. It is still an open problem to be explored, though deep learning methods have made significant progress in 2D object detection [1,2,3,4,5,6] and have a relatively unified framework. At present, 3D object detection based on point clouds has attracted more attention of researchers due to some characteristics of lidar, such as accurate depth information and high resolution. However, the point clouds scanned by lidar are usually sparse and only obtain some of the points on an object’s surface owing to occlusion, as it is challenging to encode a scene and extract robust features.

The mainstream methods can be divided into two types. One is based on multi-sensor fusion [7,8,9,10], mainly cameras and lidar. The images captured by the camera contain rich color and texture information, while the point clouds scanned by lidar contain accurate depth information. Therefore, how to effectively combine the advantages of the two is a current research hotspot, but few frameworks can effectively integrate their features elegantly. The other is based on the pure point cloud, which can be further subdivided into three methods: (1) Point-based methods [11,12,13], most of which are extensions of PointNet [14] and its variants, can extract features directly from the original point cloud, with each point having a flexible receptive field to aggregate features by set abstraction operations. In addition, they provide precise location information, but a high time cost. The second type comprises (2) voxel-based methods [15,16,17,18], which encode the entire scene into voxels and use a regular 3D CNN (convolutional neural network) to obtain more effective feature expression. This method is also very time consuming at first, but due to the introduction of sparse convolution, the calculation speed is accelerated. However, the downsampled convolutional features extracted from the point cloud will inevitably lose structure details, which are vital for generating accurate localization. The third type comprises (3) point–voxel-based methods [18,19], which combine the advantages of the previous two methods. While transforming the receiving fields flexibly to extract robust features, they can also be aware of structural information, which greatly improves the detection accuracy. These methods are dedicated to designing more effective network structures or extracting more robust feature expressions from real point clouds to obtain more objective information.

Recently, an approach called Associate-3Ddet [20] has inspired a new idea. It observes that the appearance of different objects of the same category in the point cloud scene is very different and proposes a domain-adaptation-like detection approach. Specifically, it uses objects with more points to replace objects with fewer points in the real scene to construct a conceptual scene and makes the difference between features from the real scene of the perception domain and features from the concept scene of the conceptual domain as small as possible. However, most of the above algorithms ignore the prior information that a car has symmetry. In a point cloud scene, an object is composed of a series of points on its surface, which are called foreground points. Therefore, we can easily calculate the symmetrical coordinates of these points about the car’s symmetry plane, which will be position labels for our network to predict the coordinates of the symmetry points.

In this paper, we propose a two-stage 3D detection framework called EPGNet. In the first stage, we encode the whole scene into voxels and use an encoder–decoder network to extract non-empty voxel-wise features to segment the foreground points and predict the positions of their symmetry points. In the second stage, we combine the symmetrical points and non-empty voxel centers to form an enhanced point cloud and then use an RPN (region proposal network) to generate detection results. Extensive experiments indicate that the proposed framework has a good performance on the KITTI benchmark. Our contributions can be summarized as follows:

- We propose a new task to predict the positions of symmetry points, which can complete the missing symmetry part of the object in the point cloud, so as to better detect the objects.
- We propose a simple method for calculating the symmetry point labels, that is, the position of a symmetry point in the point cloud coordinate system can be calculated indirectly through its relative position in the 3D ground truth box.
- Our detector achieves competitive detection performance in both 3D and BEV (bird’s eye view) detection and runs at 14 FPS, which is faster than many other two-stage methods.

## 2. Related Work

This section mainly introduces point cloud feature representation and two kinds of 3D object detection methods based on lidar and multiple sensors.

### 2.1. Deep Learning on Point Cloud Feature Representation

Generally, there are two ways to learn features from point clouds for 3D detection.

PointNet [14] is a pioneering effort that directly processes point sets. Aiming at the displacement invariance and rotation invariance of the point cloud, it designs a network structure to extract effective features from the original point cloud. The basic idea of PointNet is to learn the spatial coding of each point, and then aggregate all individual point features into a global point cloud signature. Therefore, it cannot capture local structural information. Its follow-up work, named PointNet++ [21], solves this problem. PointNet++ introduces a hierarchical network structure. It uses the farthest point sampling to sample some points uniformly for each layer, whose features are used as the input of the current layer, and then applies PointNet for feature extraction. Its most important innovation is the use of set abstraction to aggregate features to obtain local structural information. Many point-based methods use PointNet and its variants as the backbone for extracting features.

Two- and three-dimensional CNNs have been proved to be effective in many tasks, so some works project point clouds onto different views or divide them into voxels, and then extract features using regular 2D or 3D CNNs. There are two dilemmas in common convolution processing of sparse data. One is that it is easy to extract distortion features; the other is unnecessary calculation. Three-dimensional sparse convolution [22] is proposed to solve these problems by restricting the output sparsity according to the input sparsity. Specifically, only non-empty voxel features are convoluted, which dramatically reduces the computational cost.

### 2.2. Three-Dimensional Object Detection with Multiple Sensors

Some existing methods use multi-modal data captured by multi-sensors (e.g., lidar and cameras) as input for 3D object detection. The pioneering work of MV3D [7] and its follow-up work, AVOD [8], project point clouds onto multi-views, such as BEV (bird’s eye view) and front view, and then extract features from images and multi-view maps, which are cropped and fused by projecting 3D proposals to the corresponding 2D feature maps. The difficulty of this method is the way of fusing the two features owing to their different characteristics and distributions.

Differently, F-Pointnet [23] extracts the 3D bounding frustum of an object by extruding 2D bounding boxes from image detectors, which leads to performance that is heavily dependent on 2D detection accuracy, and then segments the foreground points to help 3D box regression in each 3D frustum. This algorithm assumes that there is only one object to be detected in the 3D frustum, which may lead to some objects going undetected.

ContFuse [9] proposes a continuous fusion layer to encode both discrete-state image features as well as continuous geometric information and to fuse multi-scale image features into point cloud features. MMF [10] proposes a new multi-sensor fusion architecture that leverages the advantages from both point-wise and RoI-wise feature fusion, resulting in fully fused feature representations. Although the above methods have certain effects, they are not simple and elegant enough.

### 2.3. Three-Dimensional Object Detection with Lidar Only

At present, more and more works are devoted to 3D object detection on pure point clouds. Although point clouds have no color or texture information, they retain richer structural information than images.

PointRCNN [11] is a milestone work of point-based methods, which is a two-stage detector. In the first stage, it applies PointNet++ to extract point-wise features and segment the foreground points for generating a small number of high-quality 3D proposals in a bottom-up manner. In the second stage, the generated proposals are converted into local coordinates, and the features of points in the proposal are aggregated for classification and regression for 3D box refinement. It has good performance, while the inference speed is relatively slow. To solve this problem, 3DSSD [13] eliminates the original up-sampling layer and refinement stage and adopts a feature fusion strategy to detect a small number of representative points, achieving a balance between speed and accuracy. STD [12] proposes a spherical anchor, which can improve the recall of the proposal.

VoxelNet [15] encodes the 3D scene into voxels and uses PointNet to extract features for points in each voxel. They are then aggregated to obtain voxel features to generate 3D boxes. Owing to the huge space of 3D scene, there will be many voxels without points, named empty voxels, which are particularly time consuming and inefficient when performing convolution operations. To solve this problem, SECOND [16] introduces sparse 3D convolution for 3D object detection, which greatly reduces the computing time. Pointpillar [24] observes that for an autonomous driving scene, the two objects will not be stacked in height, so the 3D scene is encoded into pillars and the extracted features are constructed into pseudo-images. The 2D CNNs are used for subsequent operations, which greatly improves the efficiency of feature extraction so that it can be able to detect objects in real time.

## 3. EPGNet for Point Cloud Object Detection

In this section, we first analyze the motivation of our idea and then present our proposed two-stage detection framework.

### 3.1. Motivation

As shown in Table 1, the detection level of an object is related to its size, truncation degree, and visual degree in the image. For the algorithms based on point clouds only, the levels defined by the image are undoubtedly different from the actual detection difficulty. Moreover, there is no scale problem because the point cloud can reflect the actual size of an object.

In the training data of an epoch, we count the numerical characteristics of the number of points contained in the objects with different detection levels, as shown in Table 2. We can see that even hard-level objects may contain many points. Therefore, the factor that affects the performance of the point-cloud-based algorithm may be the number of points contained in the objects. A natural idea is to increase the points of the objects, but also to ensure that the added points are distributed on the surfaces of the objects. The main concern in an autonomous driving scene is the car. Considering the symmetry of the car, we calculate the symmetrical coordinates of the foreground points about the symmetry plane of the car, and the positions of the generated symmetry points will provide us with supervision information.

**Figure 1:**
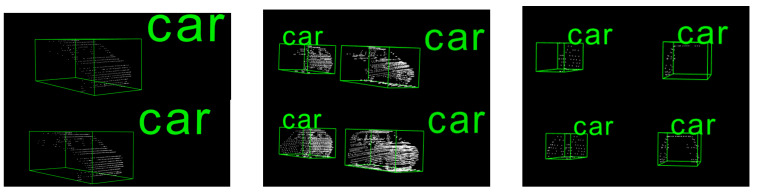
Cars with symmetry points. From **left** to **right**, the images show easy-, moderate-, and hard-level cars before and after generating symmetry points.

Specifically, we first calculate the relative position of each foreground point in the 3D ground-truth boxes according to Equation (1). We denote the relative position of a foreground point ($p_{x}$, $p_{y}$, $p_{z}$) as three continuous values ($r_{x}$, $r_{y}$, $r_{z}$). A 3D box is represented as ($C_{x}$, $C_{y}$, $C_{z}$, *w*, *l*, *h*, θ). ($C_{x}$, $C_{y}$, $C_{z}$) is the center of this 3D box, while (*w*, *l*, *h*) is the size and θ is the orientation in bird’s eye view. All coordinates are represented in the lidar coordinate system, where the direction of z is perpendicular to the ground, and x and y are parallel to the horizontal plane.

(1) $$\begin{array}{cc} \; & \left[\begin{array}{cc} t_{x} & t_{y} \end{array}\right]=\left[\begin{array}{cc} p_{x}-C_{x} & p_{y}-C_{y} \end{array}\right]\left[\begin{array}{cc} \cos(-\theta ) & -\sin(-\theta )\\ \sin(-\theta ) & \cos(-\theta ) \end{array}\right]\\ \; & \;r_{x}=\frac{t_{x}}{w}+0.5\;r_{y}=\frac{t_{y}}{l}+0.5\;r_{z}=\frac{p_{z}-C_{z}}{h}+0.5, \end{array}$$

where ($r_{x}$, $r_{y}$, $r_{z}$)∈ [0, 1] and the relative position of the object center is (0.5, 0.5, 0.5). Then, we can get the relative position of the symmetry point, denoted as ($1-r_{x}$, $r_{y}$, $r_{z}$). Finally, we substitute the relative position of the symmetry point into Equation (1) to find out the position of the symmetry point in the point cloud coordinate system, denoted as ($p_{x}^{{}^{\prime }}$, $p_{y}^{{}^{\prime }}$, $p_{z}^{{}^{\prime }}$). The cars of different levels before and after generating symmetry points are shown in Figure 1. The effectiveness of this method is also proved later in Section 4.3.1. The problem in front of us is how to let the network predict the locations of these symmetry points. We will introduce how we solve this problem below.

### 3.2. Network Architecture

**Figure 2:**
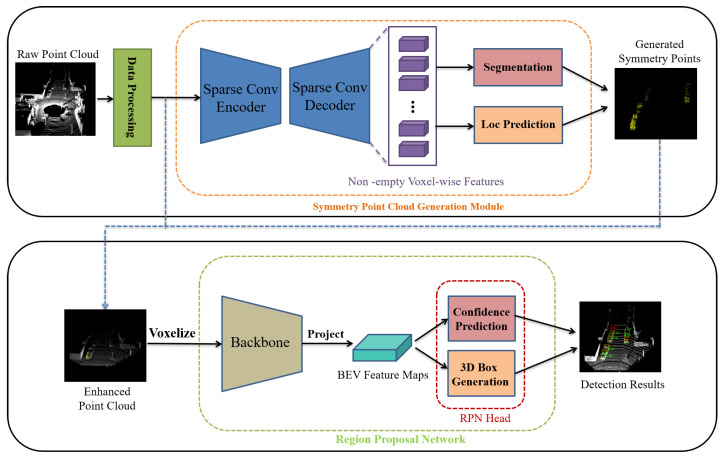
The overall architecture of our network.

The overall structure of our proposed EPGNet is illustrated in Figure 2, which consists of three parts: (1) data processing; (2) symmetry point generation module; (3) region proposal network.

In the first stage, the original point cloud is transformed into voxels through the data processing module and then input into the symmetric point generation module for foreground point segmentation and symmetry point prediction. The generated symmetry points and non-empty voxel centers form an enhanced point cloud. In the second stage, the enhanced point cloud is input into the baseline, an anchor-based region proposal network, to generate the final detection results.

#### 3.2.1. Data Processing

The space of the complete 3D point cloud scene scanned by lidar is often very large. Without any processing, direct voxelization not only consumes more time, but also occupies a lot of video memory for subsequent feature extraction. Hence, we need to limit the scene size. For a given point cloud, we only deal with the range of [0 m, −40 m, −3 m, 70.4 m, 40 m, 1 m] ([$x_{min},y_{min},z_{min},x_{max},y_{max},z_{max}$]) in the point cloud coordinate system. We also only focus on the point cloud in the field of view of the left camera. Specifically, we use the coordinate transformation matrix to project the point cloud to the left image plane and ignore the points outside the image range.

After the above series of operations on the original point cloud, we voxelized the scene with a voxel size of 0.05 m × 0.05 m × 0.1 m. So, the resolution of voxelized 3D space among the X-axis, Y-axis, and Z-axis is 1408, 1600, 40. Each point will fall into the corresponding voxel. When the points in a voxel exceed a certain number of *N*, random sampling will be conducted, leaving only *N* points. If the number is less than *N*, zero will be filled. The average coordinate values of points in non-empty voxels are used as the initial values of voxel features. The point set composed of the centers of the generated voxels can be regarded as a regular point cloud. The smaller the size of the voxel, the smaller the difference from the original point cloud. Therefore, the features of a voxel can also be regarded as the features of the voxel center.

#### 3.2.2. Symmetry Point Generation Network

**Figure 3:**
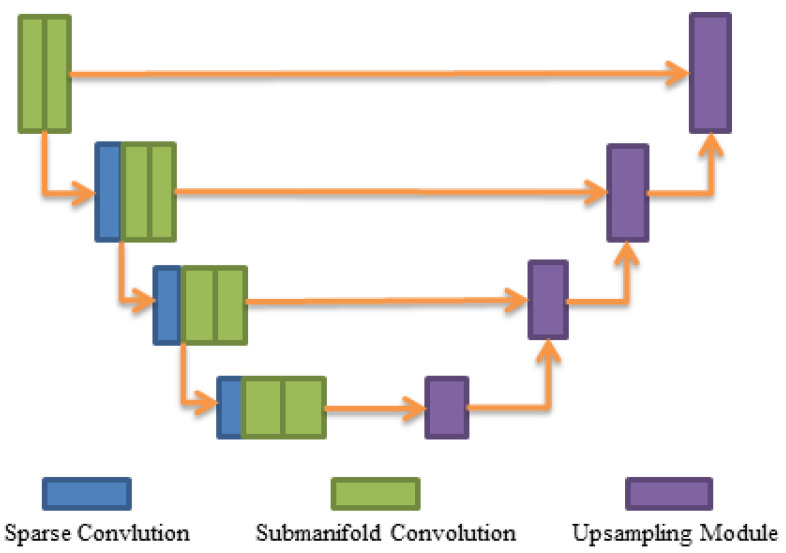
UNet-like encoder–decoder. The encoder downsamples the feature volume by eight times, and the decoder restores it to the original scale and extracts non-empty voxel-wise features.

In practical applications, we cannot directly calculate the location of a symmetry point using Equation (1) for the data that we have not labeled. So, we need a network that can segment the foreground points and predict the positions of their symmetry points. To accomplish these two tasks, we use a feature extractor similar to UNet [26] proposed by [27] to extract non-empty voxel-wise features. The network structure is shown in Figure 3, which is mainly composed of sparse convolution layers and submanifold convolution [28] layers. The encoder uses three sparse convolutions with stride 2 to downsample the spatial resolution eight times. Each sparse convolution layer is followed by two submanifold convolutions. The decoder uses four upsampling blocks to restore the spatial resolution to the original scale so that we can get effective non-empty voxel-wise feature representation. After the decoder, a segmentation head and a regression head are added to segment the point cloud and estimate the positions of the symmetry points, respectively.

The 3D ground-truth boxes naturally provide us with the semantic label of each point. The points in the boxes are considered as the foreground points, and are otherwise the background points. Considering the great gap between the number of foreground points and background points for automatic driving scenes, we use the focal loss proposed by [29] to alleviate the foreground–background class imbalance problem:

(2) $$\begin{array}{c} L_{seg}=\frac{1}{N_{pos}}\sum_{i}^{N}-\alpha_{t}{\left(1-{\hat{s}}_{i}\right)}^{\gamma }log\left({\hat{s}}_{i}\right)\\ \mathrm{where}\;{\hat{s}}_{i}=\left\{\begin{array}{cc} {\tilde{s}}_{i}, & ifs_{i}=1\\ 1-{\tilde{s}}_{i}, & \mathrm{otherwise}. \end{array}\right. \end{array}$$

where $s_{i}$ is a binary label used to indicate whether a point is a foreground point, and ${\tilde{s}}_{i}$ is the estimated probability of the network for a foreground point belonging to [0, 1]. $N_{pos}$ is the number of foreground points. $\alpha_{t}$ and γ are hyperparameters, with the same settings, $\alpha_{t}$ = 0.25 and γ = 2, as those that the original paper used in the training phase.

In Section 3.1, we have introduced the method of calculating the position label of the symmetry point, which is expressed as ($p_{x}^{{}^{\prime }}$, $p_{y}^{{}^{\prime }}$, $p_{z}^{{}^{\prime }}$). Then, we can obtain the position offsets ΔP of the symmetry point relative to its corresponding foreground point. It should be noted that a pair of symmetrical points here are in the same height plane, so only the offsets in the X and Y directions need to be calculated, and then $\Delta P=\left(p_{x}^{{}^{\prime }}-p_{x},p_{y}^{{}^{\prime }}-p_{y}\right)\in R^{2}$. Assuming that the position offset predicted by the network is $\Delta \tilde{P}=\left(\Delta \tilde{x},\Delta \tilde{y}\right)$, the loss of the position prediction branch can be represented by the following smooth-$l_{1}$ loss as

(3) $$L_{loc}=\frac{1}{N_{pos}}\sum_{i}^{N}Smooth-l_{1}\left(\Delta {\tilde{P}}_{i}-\Delta P_{i}\right)\cdot I\left[s_{i}=1\right]$$

where I[·] is a indicator function.

When the segmentation score of a point is greater than a threshold *T*, the point is considered to be a foreground point, and we can also get the position of the corresponding symmetry point estimated by the network, denoted as ($p_{x}+\Delta \tilde{x}$, $p_{y}+\Delta \tilde{y}$, $p_{z}$). Let the point set composed of these symmetry points be $X=\left\{S_{i}|i=1,2...,m\right\}$ and the point set composed of non-empty voxel centers be $Y=\left\{V_{i}|i=1,2...,n\right\}$, where $S_{i}$ is the vector of a point, which is composed of its own coordinates and additional feature channels, such as color, intensity, etc., and so is $P_{i}$. Since the intensity of the symmetry point is unknown, we only use the coordinates of the points as the initial feature in this paper. The generated symmetry points and non-empty voxel centers form an enhanced point cloud expressed as Z=X∪Y, which will be the input of the baseline.

#### 3.2.3. Region Proposal Network

**Figure 4:**
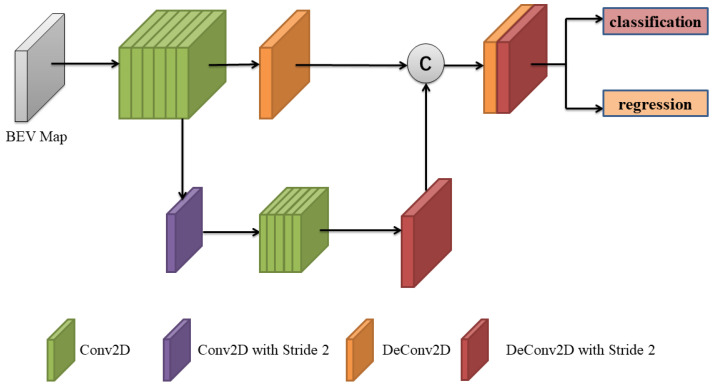
Region proposal network (RPN) head. The same-colored boxes represent the features after the convolution of the same type.

The region proposal network consists of a backbone network and a detector head. The backbone network has the same structure as the encoder in the first stage and only adds a sparse convolution after the encoder to compress the height of the feature volume. The enhanced point cloud Z is divided into voxels with a spatial resolution of H×L×W. The feature volume extracted by the backbone network is downsampled eight times, which can be expressed as a tensor form $\left[C,\frac{H}{8},\frac{L}{8},\frac{W}{8}\right]$. After that, the height channels of this feature are compressed into BEV representation, represented as $\left[C\times \frac{H}{16},\frac{L}{8},\frac{W}{8}\right]$. Then, we apply the RPN head shown in Figure 4 to 3D box classification and regression, which is similar to SSD [30].

For cars, we place anchors of two directions, $0^{\circ }$ and $90^{\circ }$, on each pixel of the BEV feature maps, so there are $2\times \frac{L}{8}\times \frac{W}{8}$ anchors, of which the size is the average size of the cars in the dataset. An IoU (intersect in union) threshold of 0.6 is used to assign the anchor to the ground truth, and if the IoU is less than 0.45, the anchor is assigned to the background. We also apply focal loss $L_{cls}$ with default parameter sets to box classification, and $smooth-L_{1}$ loss is adopted to regress the normalized box parameters:

(4) $$L_{bbox}=\sum_{r\in \left\{x,y,z,h,l,w,\theta \right\}}smooth-L_{1}\left(\Delta {\hat{r}}_{t},\Delta r_{t}\right)$$

where $\Delta {\hat{r}}_{t}$ is the predicted residual and $\Delta r_{t}$ is the regression target. The specific encoding method is as follows:

(5) $$\begin{array}{cc} \; & \Delta x_{t}=\frac{x_{g}-x_{a}}{d_{a}},\;\Delta y_{t}=\frac{y_{g}-y_{a}}{d_{a}},\;\Delta z_{t}=\frac{z_{g}-z_{a}}{h_{a}},\\ \; & \Delta w_{t}=log\left(\frac{w_{g}}{w_{a}}\right),\;\Delta l_{t}=log\left(\frac{l_{g}}{l_{a}}\right),\;\Delta h_{t}=log\left(\frac{h_{g}}{h_{a}}\right),\\ \; & \Delta \theta_{t}=\theta_{g}-\theta_{a},\;d_{a}=\sqrt{{\left(w_{a}\right)}^{2}+{\left(l_{a}\right)}^{2}} \end{array}$$

where the subscripts *a*, *g*, and *t* respectively indicate the anchor, the encoded value, and the ground truth.

#### 3.2.4. Loss Function

The loss of the first stage can be expressed as

(6) $$L_{1}=\delta L_{loc}+\zeta L_{seg}$$

The loss of the second stage can be expressed as

(7) $$L_{2}=\sigma L_{bbox}+\lambda L_{cls}$$

So, the total loss of our network can be expressed as

(8) $$L_{total}=L_{1}+L_{2}$$

where δ,ζ,σ,λ are hyperparameters to balance these losses. In this paper, they are equal to 1, 1, 2, and 1, respectively.

## 4. Experiments

Our EPGNet was evaluated on the challenging 3D and BEV KITTI object detection benchmark. We first introduce some implementation details in Section 4.1, including the dataset partitioning and parameter settings, and then compare the 3D AP (average precision) and BEV AP with some 3D object detection algorithms in Section 4.2. Furthermore, we conduct some ablation experiments in Section 4.3 to verify the effectiveness of our method. Finally, we analyze the results of symmetry point generation and object detection in Section 4.4.

### 4.1. Implementation Details

#### 4.1.1. KITTI Dataset

The KITTI benchmark was jointly created by the Karlsruhe Institute of Technology and Toyota American Institute of Technology. It is the largest computer vision algorithm evaluation dataset for automatic driving scenes in the world. It contains 7481 training samples and 7518 test samples, each of which has point cloud data and a corresponding image. Since the annotations of the test set are held by the official authorities and are not made public, we divide the training set into a training split with 3712 samples and a validation split with 3769 samples according to the common division method. All the experiments in the following content are trained with the training split and evaluated with the validation split.

#### 4.1.2. Network Details

In the first stage, the encoder uses a submanifold convolution to increase the feature dimension from 3 to 16, and then uses three sparse convolutions with kernel size 3×3×3 and stride 2×2×2 to downsample the spatial resolution eight times, with the feature dimensions being 32, 64, and 64 from top to bottom. The decoder uses four upsampling blocks to restore the spatial resolution to the original scale, noting that the stride of the top upsampling block is 1. The feature dimensions, from bottom to top, are 64, 64, 32, and 16. Each sparse convolution is followed by a BN layer and an ReLU layer. In the second stage, the backbone parameter settings of the RPN are the same as those of the encoder in the first stage, noting that the kernel size and the stride of the added sparse convolution are 3×1×1 and 2×1×1, respectively.

We also use common data augmentation, such as random flipping, global scaling with a scaling factor randomly sampled from [0.95, 1.05], and global rotation around the vertical axis by an angle sampled from [$-\frac{\pi }{4}$, $\frac{\pi }{4}$]. Referring to a “copy and paste” data augmentation method proposed by [16], we put all 3D GT boxes and the points in them into a constructed data pool. During training, we randomly selected some 3D boxes from the data pool and placed them in the current scene, noting that any two boxes cannot have an intersection.

#### 4.1.3. Training Details

During training, we used the ADAM optimizer to train our model with batch size 3 for 80 epochs, which will take about 40 h using a single GTX 1080 Ti card. For the cosine annealing learning rate strategy, the initial learning rate was set to 0.001. When the segmentation score of a point predicted by the symmetry point cloud generation module exceeds the threshold *T* = 0.7, the point is considered to be a foreground point. The size of anchors used for regressing 3D boxes of cars is 1.6 m × 3.9 m × 1.56 m.

### 4.2. Comparisons on the KITTI Validation Set

**Figure 5:**
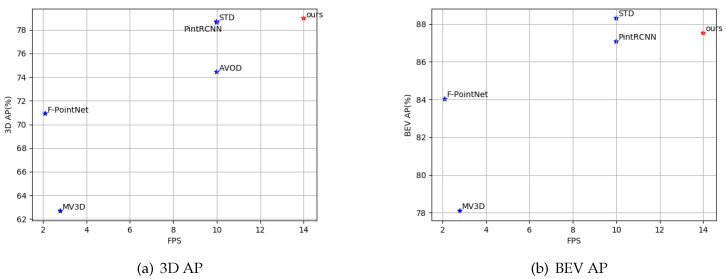
AP metric of 3D and BEV car detection at the moderate level compared with the two-stage methods in Table 3.

We compare the performance of our proposed EPGNet with some previous state-of-the-art methods on 3D and BEV object detection using the KITTI validation set. To fully compare the various methods, we chose the single-stage and two-stage methods with different modalities. At the same time, we bold the relevant values in Table 3 to highlight the optimal value of each metric. KITTI uses the moderate level of 3D object detection as the most important metric for detector performance. It can be seen from Table 3 that our EPGNet has achieved competitive results in both 3D and BEV object detection; it has reached the top in 3D object detection, and is superior to most algorithms in BEV detection. In order to show the superiority of our algorithm more intuitively, we write the APs at the moderate detection level for two-stage algorithms in Table 3 and show them in Figure 5. The horizontal axis represents the frame rate, and the vertical axis represents the AP. The closer the algorithm is to the upper right corner, the better its performance.

Although EPGNet predicts 3D-oriented boxes, the BEV and 3D metrics do not take the direction into consideration. Orientation is evaluated using average orientation similarity (AOS), which requires projecting a 3D box onto the image, performing 2D detection matches, and then evaluating the directions of these matches. Compared with the existing data, the average performance of EPGNet on AOS significantly exceeds all strata (Table 4), especially at a simple level of difficulty. Our method can make the orientation more accurate and the regression effect better.

### 4.3. Ablation Studies

#### 4.3.1. The Upper-Bound Performance of Our EPGNet

When the symmetry point generation module can accurately predict the positions of all the symmetric points, our algorithm reaches the upper-bound performance. In order to simulate this situation, we omit the symmetry point generation module, directly generate the symmetric point according to 3D box annotations and Equation (1), and form the enhanced point cloud with the original point cloud as the input of the baseline. The experimental results are given in Table 5.

Firstly, these results show that it is feasible to improve the detection results by using symmetry points. Secondly, they represent the great potential of our method, of which the upper-bound performance is far better than that of the state-of-the-art methods.

#### 4.3.2. Hyperparameters for Training and Testing

To evaluate the effects of hyperparameters for segmenting foreground points, we conducted experiments with different settings of the threshold *T*. The larger the *T*, the greater the probability that the selected points are the foreground points, and the more the corresponding symmetry points meet our requirements. However, it is also more likely to miss some foreground points. The results are shown in Table 6.

We can see from Table 6a that setting $T_{train}$ to 0.7 works best. If $T_{train}$ is set to a lower value during training, it will lead to performance degradation because a lot of background points will be introduced. Table 6b shows the results of different thresholds $T_{eval}$ for testing at $T_{train}=0.7$. We can conclude that when the training threshold $T_{train}$ is fixed, and the evaluating threshold $T_{eval}$ is greater than the training threshold $T_{train}$, AP will decrease. On the contrary, it will increase. The lower the evaluating threshold, the more points are introduced, including useful symmetry points and useless noise points. However, if our model uses a higher training threshold, even if there are more noise points during evaluation, it will not interfere with the detection of the model. The comparison shows that setting $T_{eval}$ to 0.3 achieves superior performance on AP3d at the moderate level.

#### 4.3.3. Location Prediction Loss

The precise positions of the symmetry points are critical to the subsequent 3D box regression, so it is necessary to choose a suitable position loss regression function. We used smooth-$l_{1}$ loss and cross-entropy loss for comparison. Specifically, the predicted position of a symmetry point can be expressed as $\left(p_{x}+\Delta \tilde{x},p_{y}+\Delta \tilde{y},p_{z}\right)$, and then the relative position $\left(\tilde{r_{x}},\tilde{r_{y}},r_{z}\right)$ of the predicted symmetry point is calculated using Equation (1). So, the cross-entropy loss function can be expressed as

(9) $$L_{loc}=-\left(r_{u}log\left(\tilde{r_{u}}\right)+\left(1-r_{u}\right)log\left(1-\tilde{r_{u}}\right)\right),$$

where u∈x,y. The comparison results are shown in Table 7. The overall effect of smooth-$l_{1}$ is better than cross-entropy loss.

### 4.4. Analysis of the Results

**Figure 6:**
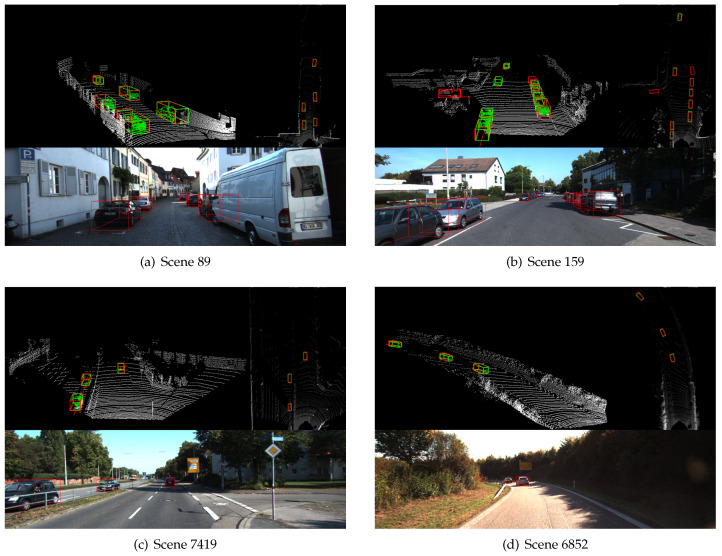
Detection results. Each scene consists of three sub-images, including a 3D point cloud (**upper left**), a BEV map (**upper right**), and a corresponding image (**below**). The red boxes represent the detection results and the green boxes represent the ground truth.

Figure 6 shows some detection results, from which we can see that our network can accurately detect even heavily obscured and far-away cars. In order to fully reflect the superiority of our algorithm, we use the second stage as the baseline and compare it with our network in the following four aspects.

#### 4.4.1. Detection of More Cars

**Figure 7:**
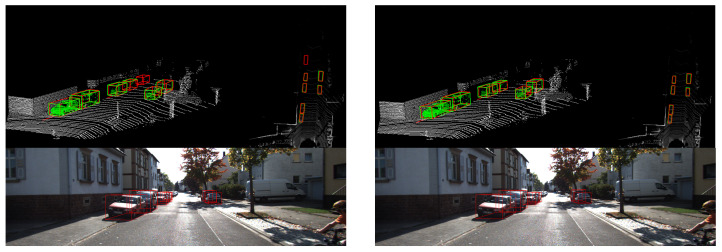
Detection results with our network (**left**) and the baseline (**right**).

As we know, the KITTI dataset ignores objects that are too far away from the lidar or that are seriously occluded when labeling data. As shown in Figure 7, although the baseline performance is excellent, our network performance is even better, as unlabeled cars can be detected.

#### 4.4.2. Less False Detection

**Figure 8:**
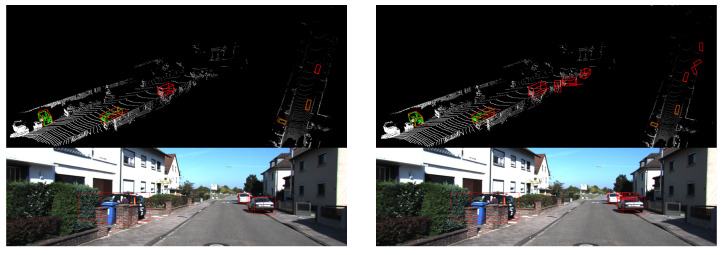
Comparison of error detection between our network (**left**) and the baseline (**right**).

Since the point cloud only contains shape information and lacks color and texture information, the network is likely to mistakenly identify some point clouds with structures similar to cars as cars, just like the baseline detection results in Figure 8. However, our network can segment the foreground points and predict positions of their symmetry points, so we can obtain more structural information to avoid some of such false detections.

#### 4.4.3. Better Regression

**Figure 9:**
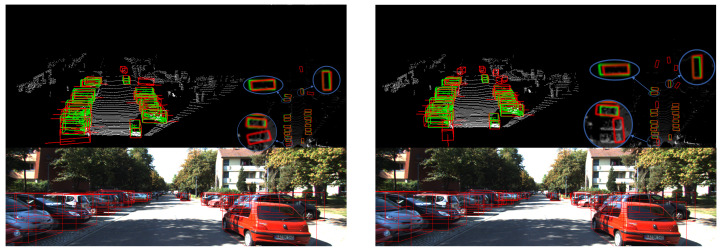
The regression effect of our network (**left**) and the baseline (**right**). Some of the details are circled and magnified.

Thanks to the generated symmetry points, our network can better regress the 3D boxes of the enveloping objects. Figure 9 shows the superiority of our method, which can not only regress the orientation correctly, but also makes the error between the detection box and ground truth smaller.

#### 4.4.4. High-Quality Symmetry Point Generation

**Figure 10:**
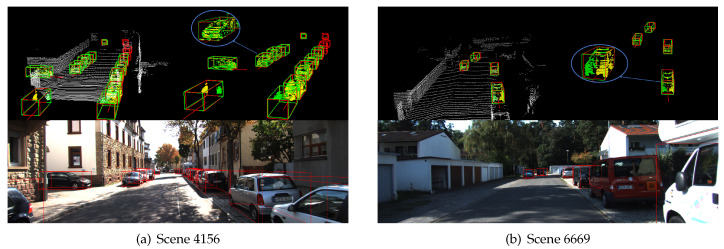
Symmetry point generation results. The symmetry points are yellow, while the foreground points are green.

As shown in Figure 10, benefiting from the foreground point segmentation task and symmetry point prediction task, our network can accurately estimate the locations of symmetry points corresponding to the foreground points so that we can obtain more objective information.

## 5. Discussion

Our method is not only suitable for car detection, but also for detection of various symmetrical objects in a point cloud scene. At the same time, it also supports replacing the baseline in the second stage with some other voxel-based single-stage methods. Of course, it also has limitations. In Table 5, we give the upper-bound performance of this method, but as seen in the final test results, there is still a lot of room for improvement. This is mainly due to the fact that most of the symmetry points generated in the first stage belong to objects with more points, and the points of objects with fewer points are difficult to segment. When the threshold *T* is set to be large, the symmetry points of these points will be filtered out. Moreover, for the automatic driving scene, our method does not support pedestrian detection at present because their postures will lead to the failure to generate the correct symmetry point labels. Future research directions should focus on how to segment the difficult points, such as designing more effective network structures and considering multi-sensor fusion. Of course, we will also try to use a method similar to domain adaptation, such as Associate-3Ddet [20].

## 6. Conclusions

In this paper, we propose a two-stage detection framework called EPGNet. In the first stage, the network segmented the foreground points and predicted positions of their symmetry points. The symmetry points and the original point cloud form an enhanced point cloud as the input of the second stage, and the objects are detected through a region proposal network. A large number of experiments on the KITTI benchmark verified the effectiveness of our method, which can accurately predict the positions of the symmetry points and detect the objects.

## Figures and Tables

**Table 1:** Definition of different detection difficulty levels on the KITTI benchmark [25].

Difficulty	Min Box Height/Pixel	Max Occlusion	Max Truncation
Easy	40	Full visible	15%
Moderate	25	Partly occluded	30%
Hard	25	Difficult to see	30%

**Table 2:** The numerical characteristics of the number of points contained in the 3D groud-truth boxes with different detection levels in the training data of an epoch. Num denotes the number of samples with the same level, and the value in parentheses denotes the number of occurrences.

Difficulty	Min	Max	Average	Median	Mode	Num
Easy	0	4378	426	256	116 (20)	4836
Moderate	0	3895	197	66	34 (83)	6173
Hard	0	4295	153	51	0 (107)	3532

**Table 3:** Performance on 3D and bird’s eye view (BEV) object detection using the KITTI validation split set at intersection in union (IoU) = 0.7 for cars. The average precision (AP) is calculated with 11 recall positions.

Scheme	Method	Modality	3D (%)			BEV (%)			FPS
			Easy	Mod	Hard	Easy	Mod	Hard	
one-stage	VoxelNet [15]	LIDAR	81.98	65.46	62.85	89.60	84.81	78.57	4.4
	SECOND [16]	LIDAR	87.43	76.48	69.10	89.96	87.07	79.66	25
	PointPillars [24]	LIDAR	86.13	77.03	72.43	89.93	86.92	84.97	62
	Voxel-FPN [17]	LIDAR	88.27	77.86	75.84	90.28	87.92	86.27	50
two-stage	MV3D [7]	RGB + LIDAR	71.29	62.68	56.56	86.55	78.10	76.67	2.8
	AVOD [8]	RGB + LIDAR	84.41	74.44	68.65	-	-	-	10
	F-PointNet [23]	RGB + LIDAR	83.76	70.92	63.65	88.16	84.02	76.44	2.1
	PointRCNN [11]	LIDAR	88.88	78.63	77.38	89.96	87.07	79.66	10
	STD [12]	LIDAR	88.80	78.70	**78.20**	90.10	**88.30**	**87.40**	10
	ours	LIDAR	**89.30**	**78.98**	77.79	**90.32**	87.52	86.02	14

**Table 4:** Results of the average orientation similarity (AOS) detection with the KITTI benchmark.

Method	mAOS	AOS (%)		
	Moderate	Easy	Moderate	Hard
SECOND	80.37	87.84	81.31	71.95
AVOD-FPN	85.61	89.95	87.13	79.74
PointPillar	88.44	90.19	88.76	86.38
ours	**90.67/+ 2.23**	**94.58/+ 4.39**	**89.18/+ 0.42**	**88.25/+ 1.87**

**Table 5:** The upper-bound performance of EPGNet with the enhanced data on AP3d.

Training Data	Validation Data	AP (IoU = 0.7)		
		Easy	Moderate	Hard
enhance	original	44.56	31.39	26.44
original	enhance	89.73	84.01	84.35
original	original	88.48	78.14	76.68
enhance	enhance	**90.33**	**89.09**	**89.11**

**Table 6:** Influence of different *T* for training and evaluation.

(a) Influence of Different $T_{train}$ on AP3d.			
$T_{train}$	AP3d (%)		
	Easy	Moderate	Hard
0.9	88.45	78.42	77.16
0.8	88.85	78.66	77.15
0.7	89.01	**78.78**	**77.32**
0.6	88.88	78.43	76.89
0.5	**89.35**	78.67	76.91
0.4	88.97	78.66	77.28
0.3	88.85	78.47	77.19
0.2	88.72	78.36	77.04
0.1	88.69	78.32	77.03
**(b) Influence of Different $T_{eval}$ on AP3d when $T_{train}$ = 0.7.**			
$T_{eval}$	AP3d (%)		
	Easy	Moderate	Hard
0.9	88.56	78.52	76.55
0.8	89.13	78.85	77.14
0.7	89.01	78.78	77.32
0.6	89.14	78.87	77.41
0.5	89.14	78.88	77.51
0.4	89.25	78.96	77.63
0.3	89.30	**78.98**	77.79
0.2	89.33	78.93	**77.83**
0.1	**89.34**	78.84	77.67

**Table 7:** A comparison of different location prediction losses for 3D and BEV detection.

Loss	3D (%)			BEV (%)		
	Easy	Moderate	Hard	Easy	Moderate	Hard
Smooth-$l_{1}$ Loss	**89.30**	**78.98**	**77.79**	**90.32**	**87.52**	86.02
Cross-Entropy Loss	88.23	78.36	77.23	89.92	87.13	**86.27**
